# Linkage of cDNA expression profiles of mesencephalic dopaminergic neurons to a genome-wide in situ hybridization database

**DOI:** 10.1186/1750-1326-4-6

**Published:** 2009-01-29

**Authors:** Kambiz N Alavian, Horst H Simon

**Affiliations:** 1 Interdisciplinary Centre for Neuroscience (IZN) – Ruprecht Karls Universität 69120 Heidelberg, Germany; 2 Harvard Medical School, Neuroregeneration Labs, MRC 1, McLean Hospital, 115 Mill St, Belmont, MA 02478, USA

## Abstract

Midbrain dopaminergic neurons are involved in control of emotion, motivation and motor behavior. The loss of one of the subpopulations, substantia nigra pars compacta, is the pathological hallmark of one of the most prominent neurological disorders, Parkinson's disease. Several groups have looked at the molecular identity of midbrain dopaminergic neurons and have suggested the gene expression profile of these neurons. Here, after determining the efficiency of each screen, we provide a linked database of the genes, expressed in this neuronal population, by combining and comparing the results of six previous studies and verification of expression of each gene in dopaminergic neurons, using the collection of in situ hybridization in the Allen Brain Atlas.

## Background

The population of dopaminergic (DA) neurons in the ventral midbrain is divided into three distinct groups, substantia nigra pars compacta (SNpc) or the A9 group, ventral tegmental area (VTA) or the A10 group, and retrorubral field. The A9 and A10 groups are the main source of dopamine in the central nervous system and give rise to two major dopaminergic pathways. Neurons of the VTA innervate the nucleus accumbens and olfactory tubercle, comprising the mesolimbic and mesocortical pathways, and the target for SNpc innervation is the dorsal striatum, thereby constituting the nigrostriatal pathway [1]. These three pathways are involved in control of emotion, motivation and motor behavior and are connected to neurological conditions such as addiction, schizophrenia and to the second most common neurodegenerative disorder, Parkinson's disease (PD) [2,3]. The main pathological characteristic of PD is the progressive degeneration of DA neurons in the SNpc. The functional and pathological relevance of the neurons in SNpc and VTA necessitates in depth study of their gene expression profile. In recent years, several such studies have been carried out to identify the genes, expressed in these neuronal populations [4-9].

Allen Brain Atlas (ABA) is a large-scale collection of gene expression patterns, using high throughput, semi-automated in situ hybridization (ISH) on mouse brain sections. The expression patterns of over 21,000 genes have been uploaded so far. Taking a genomic style approach, ABA shows above background expression, in different regions of the brain, for approximately 80% of the genes. The ISH data is searchable either by anatomical structure or using a finer (100–300 μm) resolution geometric grid [10]. The data is also available as three dimensional images and graphs, through the usage of a web browser and Brain Explorer. Another function of ABA, Neuroblast, allows identification of genes with similar expression patterns within the ABA dataset [11]. In this study, we have combined/compared results of the six studies, aiming at a comprehensive expression profile of mesDA neurons, and verified the expression of the identified genes, using the digital atlas made publicly accessible by the ABA. Here, we present a database of this verification effort.

## Results

Previously, six studies have analyzed the expression profile of ventral midbrain cells or mesDA neurons, specifically, resulting in a list of several hundred genes [4-9]. Three of the studies were differential display based, which studied the expression profile of the midbrain tissue in mice. The other three studies used a more specific approach and identified the global expression profile of dopaminergic neurons of the subpopulations of mesDA neurons in rats, using microarray. In order to combine the lists of genes from the six studies, as a first step, we determined the association of sequences in the cDNA differential display-based studies (Stewart et al., Barrett et al. and Thuret et al.) to genes and published ESTs, by checking location of each hit on the mouse genome on Map Viewer  and by performing BLAST search. Among 1435 cDNA fragments, 1050 were unambiguous murine genomic hits, 19 were ambiguous multiple hits and 104 were alignments with mitochondrial DNA. 262 cDNA sequences produced no significant alignments. Then, in order to compare all six studies and make a comprehensive list, we determined the mouse accession numbers of the genes from Chung et al., Greene et al. and Grimm et al. by performing BLAST searches at . From all the genes, presented in these screens, only the accession numbers of three genes were unidentifiable. A comparison of the six screens [4-9], and elimination of the redundancies of genes, common to two or more studies, resulted in a total of 609 genes.

In order to determine whether the genes, presented in these screens, are present in VTA and/or SNpc, we looked for above-background cellular expression in the ventral midbrain, using Allen's Brain Atlas ISH database at . As a control, the expression pattern of the rate-limiting enzyme in production of dopamine, tyrosine hydroxylase (Th) [12], in coronal and sagittal sections was used. Among 609 genes, 335 (56%) showed strong expression in the SNpc or VTA (see Additional file 1). The efficiency of each screen, in identifying mesDA-specific genes was 25% for Chung et al., 29% for Barrett et al., 28% for Stewart et al., 37% for Greene et al., 24% for Thuret et al. and 24% for Grimm et al. (see Additional file 2), which is an indication for the complementary nature of the six screens. The final list of genes is presented in a database, linked to the ISH images of ABA (database available upon request).

**Figure 1:**
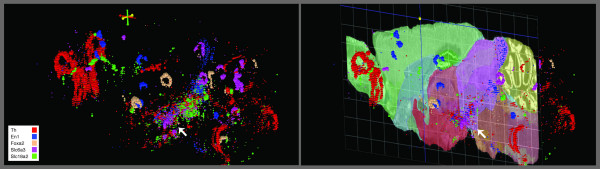
**Expression pattern of known mesDA neuron-specific transcription factors and genes involved in dopamine neurotransmission in mouse brain**. Three-dimensional view of Foxa2, En1, Th, Vmat2 (Slc18a2) and Dat (Slc6a3) expressions in the brain, superimposed on a sagittal nissl image from Allen Brain Atlas (ABA) are shown. The genes chosen for Neuroblast search have known functions in the adult mesDA neurons and are detected by ABA in ventral midbrain (arrow).

The Neuroblast function in the ABA is used to identify genes with common expression patterns in the brain or within specific regions of the brain. We used this tool to confirm our data and to determine its efficiency in identifying the expression of genes, within mesDA neuronal population. Therefore, first we performed Neuroblast to identify the list of genes with expression patterns, similar to dopaminergic neuron-specific genes, involved in the neurotransmitter phenotype of the mesDA neurons or the transcription factors, which are required for development, survival and maintenance of dopaminergic neurons in developing embryos as well as the adult organisms. Th [12], dopamine transporter (Dat), which is responsible for uptake of dopamine, vesicular monoamine transporter 2 (VMAT2), responsible for transport of dopamine into synaptic vesicles [13], Homeobox transcription factor, Engrailed-1 (En1), [14] and the forkhead transcription factor (Foxa2), [15] which are required for long term survival and maintenance of dopaminergic neurons were chosen as the base genes for the Neuroblast search. Expression of all five genes was observed in ABA coronal or sagittal series and three dimensional visualizer (Figure 1). The Neuroblast search revealed 703 genes (see Additional file 3), with similar expression patterns to one or more of the five above genes (Th, Vmat2, Dat, En1, Foxa2), of which, 70 were among the genes, expressed in mesDA neurons, according to the original six transcriptome analysis studies (see Additional file 4).

## Discussion

In this work we present a searchable database of genes, expressed by mesDA neurons, based on the results of six previous expression screens and the in situ hybridization data presented in Allen Institute's brain atlas. The efficiency of each individual screen does not exceed 37%, i.e. a large number of the genes, suggested by differential display or microarray studies, does not show above background levels of expression, detectable by ISH. However, the combination of results from all screens increases the efficiency to 56%, indicating the complementary nature of the studies.

The Neuroblast function in ABA may seem self-sufficient in determining the expression profile of a cell population within the brain, by use of the genes with high expression level within that cell population as template, but our study shows that a combination of studies such as the six, mentioned in this work, are still essential. The Neuroblast function is only partially effective, since less than 20% of the genes, identified by our approach were among the list of genes, compiled from five Neuroblast searches.

## Conclusion

From comparison of six previously published data sets and the in situ hybridization data, available at Allen Brain Atlas, we conclude a comprehensive database of the gene expression profile of midbrain dopaminergic neurons, known up to date. This database provides a starting point for further investigations in the field, attempting to identify the molecular cascades, which may result in degeneration of this neuronal population during the course of Parkinson's disease.

## Methods

All nucleotide sequences used in this study are publicly available at: . To determine whether the EST sequences, presented by the cDNA differential display-based data, i.e. Barrett et al. (779 sequences; accession numbers: BE824469 to BE824504, BE824506 to BE824519, BE824521 to BE824561, BE824563 to BE824823, BE824825 to BE825045, BE825047 to BE825132, CK338036 to CK338155), Stewart et al. (496 cDNA sequences; accession numbers: AA008736, W33210 to W33212, W33214 to W33289, W35421 to W35480, W36130 to W36269, W39787 to W40005, W40007 to W40008, W40010 to W40023, W45732) and Thuret et al. (159 sequences; accession numbers: CO436137 to CO436293) were associated to any identified gene, we checked the sequences on the Map Viewer at the NCBI website, at  and employed each nucleotide sequence for a nucleotide-nucleotide BLAST (blastn) (basic local alignment search tool) on the non-redundant database  and on the mouse genome .

In order to homogenize, compare and contrast the data among all six screens, the mouse accession numbers of the genes from the three latter studies, Chung et al., Greene et al. and Grimm et al., was obtained by using BLAST search. Then, a comparison of the screens [4-7,9,16] and elimination of the genes, common to two or more studies was carried out. In order to determine whether the genes, presented in these screens, are expressed within VTA and/or SNpc, we searched for above-background cellular expression in the ventral midbrain, using the Allen Brain Atlas at . We did not differentiate between the two populations, because the coronal images were not available for about a third of the genes. Finally, the same method was used to determine the expression of a randomly chosen set of 300 genes in the midbrain.

## Competing interests

The authors declare that they have no competing interests.

## Authors' contributions

KNA and HHS conceived of the study, carried out the analyses and drafted the manuscript. Both authors read and approved the final manuscript.

## Supplementary Material

Additional file 1 **List of genes, expressed within dopaminergic neurons of the midbrain, according to ABA**. The list of genes identified by the six screens (Chung et al., Barrett et al., Stewart et al., Greene et al., Thuret et al. and Grimm et al.), after eliminating the duplicates and triplicates, which showed expression patterns similar to tyrosine hydroxylase within ventral midbrain. Click here for file

Additional file 2 **Number of genes, identified by six screens, aimed at determining the expression profile of mesDA neurons**. Total number of genes, as well as the number of transcripts, expressed in SNpc or VTA, detectable by ISH, within the ABA database is shown. Efficiency of each screen in identifying transcripts, which show above-background expression within either or both of the two regions, according to ABA is between 24–38%, while 56% of the genes from combination of all screens show expression. Click here for file

Additional file 3 **Compilation of gene symbols, identified by Neuroblast function of ABA**. The template genes Foxa2, En1, Th, Vmat2 and Dat, used for neurblast, are expressed and have defined functions in neurotransmitter phenotype or transcriptional regulation of survival and maintenance of dopaminergic neurons. 250 genes were listed as having similar expression patterns to each template. Repetitions are eliminated from the final list. Click here for file

Additional file 4 **Comparison of the list of genes from Neuroblast and genome-wide screens**. The comparison of the two lists from additional files 1 &2 show that a fraction of the genes from the six screens, considered by this study can be acquired, using the Neuroblast function. Click here for file
