# Supplementary material for: Linkage of cDNA expression profiles of mesencephalic dopaminergic neurons to a genome-wide in situ hybridization database
Source: Mol Neurodegener. 2009 Jan 29;4:6. doi: 10.1186/1750-1326-4-6 (PMC2637272; doi:10.1186/1750-1326-4-6)
Supplement: Additional file 1 — List of genes, expressed within dopaminergic neurons of the midbrain, according to ABA. The list of genes identified by the six screens (Chung et al., Barrett et al., Stewart et al., Greene et al., Thuret et al. and Grimm et al.), after eliminating the duplicates and triplicates, which showed expression patterns similar to tyrosine hydroxylase within ventral midbrain. [file 1750-1326-4-6-S1.pdf]

Gene Name	Gene Symbol	Serial #	Screen	Accession #
myosin X	Myo10	37	Thuret	NM_019472
actin, beta, cytoplasmic	Actb	44	Thuret	NM_007393
in locus of RIKEN cDNA C130076O07 gene	C130076O07Rik	58	Thuret	
complexin I	Cplx1	64	Thuret	NM_007756
in locus of contactin associated protein 4 (Cntnap4)	Cntnap4	66	Thuret	NM_130457
similar to Ac1147	LOC385068	95	Thuret	
myeloid/lymphoid or mixed-lineage leukemia 1	Mll1	100	Thuret	XM_110671
morf4 family associated protein 1	Mrfap1	104	Thuret	NM_026242
pyridoxal (pyridoxine, vitamin B6) phosphatase	Pdxp	118	Thuret	NM_020271
polo-like kinase 1 (Drosophila)	Plk1	123	Thuret	NM_011121
Rap2 interacting protein	Rap2ip	127	Thuret	
RAN GTPase activating protein 1	Rangap1	128	Thuret	NM_011241
roundabout homolog 1 (Drosophila)	Robo1	136	Thuret	NM_019413
signal-induced proliferation-associated 1 like 1	Sipa1l1	145	Thuret	NM_172579
SWI/SNF related, matrix associated, actin dependent regulator of chrom	smarca2	150	Thuret	NM_011416
synaptotagmin 1	Syt1	155	Thuret	NM_009306
thymidine kinase 2, mitochondrial	Tk2	161	Thuret	NM_021028
	4930431J08Rik	283	Thuret	
	5830434P21Rik	284	Thuret	
insulin-like growth factor binding protein-like 1	Igfbpl1	375	Thuret	NM_018741
	A230057G18Rik	393	Thuret	
	Azi2	394	Thuret	
hydroxysteroid (17-beta) dehydrogenase 12	Hsd17b12	3	Steward	NM_019657
Bcl2-associated athanogene 1	Bag1	24	Steward	NM_009736
RIKEN cDNA 1110059H15 gene	1110059H15Rik	38	Steward	XM_981951
transmembrane protein 66	Tmem66	39	Steward	NM_026432
OTU domain containing 6B	Otud6b	42	Steward	NM_152812
acyl-CoA synthetase long-chain family member 3	Acsl3	46	Steward	NM_001033606
ARP2 actin-related protein 2 homolog	Actr2	47	Steward	NM_146243
ADP-ribosylation factor guanine nucleotide-exchange factor 2 (brefeldin	Arfgef2	50	Steward	XM_130646
ATPase, H+ transporting, lysosomal 16kD, V0 subunit C	Atp6v0c	51	Steward	NM_009729
ATPase, H+ transporting, V1 subunit E isoform 1	Atp6v1e1	52	Steward	NM_007510
ATPase, H+ transporting, V1 subunit A, isoform 1	Atp6v1a	53	Steward	NM_007508

RIKEN cDNA B430201A12 gene	B430201A12Rik	54	Steward	XM_283903
RIKEN cDNA B630019K06 gene	B630019K06Rik	55	Steward	NM_175327
cDNA sequence BC003498	Psd3	56	Steward	NM_030263
calmodulin 2	Calm2	59	Steward	NM_007589
calmodulin 3	Calm3	60	Steward	NM_007590
integrin alpha FG-GAP repeat containing 1	Itfg1	61	Steward	NM_028007
cholinergic receptor, nicotinic, beta polypeptide 3	Chrnb3	63	Steward	NM_027454
coatomer protein complex, subunit gamma 2	Copg2	65	Steward	NM_017478
TSPY-like 4	Tspyl4	67	Steward	
DEAD (aspartate-glutamate-alanine-aspartate) box polypeptide 5	Ddx5	72	Steward	NM_007840
dispatched homolog 2	Disp2	73	Steward	NM_170593
v-erb-a erythroblastic leukemia viral oncogene homolog 4 (avian)	Erbb4	77	Steward	XM_001002143
in locus of gene model 528, (NCBI)	Gm528	85	Steward	XM_147716
guanine nucleotide binding protein, beta 1	Gnb1	86	Steward	NM_008142
imprinted and ancient	Impact	89	Steward	NM_008378
kinesin family member 1A	Kif1a	90	Steward	NM_008440
kelch-like 9 (Drosophila)	Klhl9	91	Steward	NM_172871
large tumor suppressor 2	Lats2	92	Steward	NM_015771
LON peptidase N-terminal domain and ring finger 2	Lonrf2	93	Steward	NM_001029878
microtubule-associated protein, RP/EB family, member 1	Mapre1	98	Steward	NM_007896
microtubule-associated protein 1A	Mtap1a	103	Steward	XM_194040
microtubule-associated protein 4	Mtap4	106	Steward	NM_008633
21 (CDKN1A)-activated kinase 1	Pak1	116	Steward	NM_011035
protocadherin 10	Pcdh10	117	Steward	NM_011043
protein kinase inhibitor, alpha	Pkia	122	Steward	NM_008862
protein kinase, cAMP dependent, catalytic, beta	Prkacb	126	Steward	NM_011100
reticulocalbin 2	Rcn2	129	Steward	NM_011992
ras homolog gene family, member A	Rhoa	132	Steward	NM_016802
roundabout homolog 2 (Drosophila)	Robo2	135	Steward	NM_175549
SAC1 (suppressor of actin mutations 1, homolog)-like (S. cerevisiae)	Sacm1l	137	Steward	NM_030692
stearoyl-Coenzyme A desaturase 1	Scd1	140	Steward	NM_009127
SEC23A (S. cerevisiae)	Sec23a	142	Steward	NM_009147
sema domain, transmembrane domain (TM), and cytoplasmic domain, (s	Sema6d	144	Steward	NM_172537
solute carrier family 18 (vesicular monoamine), member 2	Slc18a2	146	Steward	NM_172523
solute carrier family 30 (zinc transporter), member 4	Slc30a4	147	Steward	NM_011774
solute carrier family 4, sodium bicarbonate cotransporter-like, member 1	Slc4a10	149	Steward	NM_033552

synuclein, alpha	SNCA	151	Steward	NM_001042451
synaptosomal-associated protein 91	Snap91	152	Steward	NM_013669
synaptic vesicle glycoprotein 2 b	Sv2b	154	Steward	NM_153579
synaptogyrin 3	Syngr3	156	Steward	NM_011522
synaptotagmin 4	Syt4	157	Steward	NM_009308
Tax1 (human T-cell leukemia virus type I) binding protein 1	Tax1bp1	158	Steward	NM_025816
thymosin, beta 4, X chromosome	Tmsb4x	163	Steward	NM_021278
thymosin, beta 10	Tmsb10	164	Steward	NM_025284
UDP-glucose pyrophosphorylase 2	Ugp2	166	Steward	NM_139297
3-monooxygenase/tryptophan 5-monooxygenase activation protein, gan	Ywhag	170	Steward	NM_018871
	Cxx1a	392	Steward	
aldo-keto reductase family 1, member B3	Akr1b3	1	Grimm	NM_009658
cholinergic receptor, nicotinic, alpha polypeptide 4	Chrna4	9	Grimm	NM_015730
glial cell line derived neurotrophic factor family receptor alpha 1	Gfra1	16	Grimm	NM_010279
tyrosine hydroxylase	Th	260	Grimm	NM_009377
tyrosine hydroxylase	Th	261	Grimm	NM_009377
peripherin 1	Prph1	307	Grimm	NM_013639
dopa decarboxylase	Ddc	308	Grimm	NM_016672
pterin 4 alpha carbinolamine dehydratase/dimerization cofactor of hepat	Pcbd1	309	Grimm	NM_025273
cell growth regulator with EF hand domain 1	Cgref1	310	Grimm	XM_181420
dopamine receptor D1 interacting protein	Drd1ip	311	Grimm	NM_026769
protein arginine N-methyltransferase 2	Prmt2	312	Grimm	NM_133182
TSPY-like 2	Tspyl2	313	Grimm	NM_029836
aldehyde dehydrogenase family 1, subfamily A1	Aldh1a1	314	Grimm	NM_013467
Down syndrome critical region gene 1-like 1	Dscr1l1	315	Grimm	NM_030598
RNA binding motif, single stranded interacting protein	Rbms3	316	Grimm	NM_178660
protein kinase C, alpha	Prkca	317	Grimm	NM_011101
RIKEN cDNA 2010011I20 gene	2010011I20Rik	318	Grimm	NM_025912
Ly6/Plaur domain containing 1	Lypd1	319	Grimm	NM_145100
solute carrier family 39 (zinc transporter), member 4	Slc39a4	320	Grimm	NM_028064
serine (or cysteine) peptidase inhibitor, clade B, member 6a	Serpinb6a	321	Grimm	NM_009254
NEL-like 2 (chicken)	Nell2	322	Grimm	NM_016743
potassium voltage-gated channel, Shal-related family, member 3	Kcnd3	323	Grimm	NM_001039347
protein kinase C, epsilon	Prkce	325	Grimm	NM_011104
leucine rich repeat containing 3B	Lrrc3b	326	Grimm	NM_146052
serine/threonine kinase 39, STE20/SPS1 homolog (yeast)	Stk39	327	Grimm	NM_016866

	A030009H04Rik	328	Grimm	
DNA segment, Chr 14, ERATO Doi 171, expressed	D14Ertd171e	329	Grimm	NM_177814
glucan (1,4-alpha-), branching enzyme 1	Gbe1	330	Grimm	NM_028803
dehydrogenase/reductase (SDR family) member 3	Dhrs3	331	Grimm	NM_011303
kelch-like 13 (Drosophila)	Klhl13	332	Grimm	NM_026167
aurora kinase A	Aurka	333	Grimm	NM_011497
LIM domain only 4	Lmo4	334	Grimm	NM_010723
phospholipase C, beta 1	Plcb1	335	Grimm	NM_019677
cytochrome b-561	Cyb561	336	Grimm	NM_007805
CUG triplet repeat, RNA binding protein 2	Cugbp2	337	Grimm	NM_010160
latrophilin 2	Lphn2	338	Grimm	XM_888040
protein tyrosine phosphatase, receptor type, U	Ptpru	340	Grimm	NM_011214
	BC065085	341	Grimm	
solute carrier organic anion transporter family, member 4a1	Slco4a1	342	Grimm	NM_148933
cadherin 13	Cdh13	343	Grimm	NM_019707
serine (or cysteine) peptidase inhibitor, clade E, member 2	Serpine2	344	Grimm	NM_009255
	Plekha7	345	Grimm	
angiomotin-like 1	Amotl1	348	Grimm	XM_919481
pre B-cell leukemia transcription factor 1	Pbx1	349	Grimm	NM_008783
ets variant gene 5	Etv5	351	Grimm	NM_023794
	D330050I23Rik	352	Grimm	
Purkinje cell protein 4-like 1	Pcp4l1	353	Grimm	XM_484933
endothelial differentiation, lysophosphatidic acid G-protein-coupled recep	Edg2	354	Grimm	NM_010336
teashirt zinc finger family member 1	Tshz1	355	Grimm	XM_888252
glutamate receptor interacting protein 2	Grip2	356	Grimm	XM_001000130
par-3 (partitioning defective 3) homolog (C. elegans)	Pard3	357	Grimm	NM_001013580
Ras association (RalGDS/AF-6) domain family 6	Rassf6	358	Grimm	NM_028478
eyes absent 2 homolog (Drosophila)	Eya2	359	Grimm	NM_010165
	2700045P11Rik	360	Grimm	
AT motif binding factor 1	Atbf1	361	Grimm	NM_007496
solute carrier family 31, member 1	Slc31a1	362	Grimm	NM_175090
rhodopsin	Rho	363	Grimm	NM_145383
glycoprotein (transmembrane) nmb	Gpnmb	364	Grimm	NM_053110
Ca2+-dependent activator protein for secretion 2	Cadps2	365	Grimm	NM_153163
fatty acid desaturase 3	Fads3	366	Grimm	NM_021890
calcium-sensing receptor	Casr	368	Grimm	NM_013803

transmembrane inner ear	Tmie	370	Grimm	NM_146260
glutathione peroxidase 3	Gpx3	371	Grimm	NM_008161
RIKEN cDNA A930038C07 gene	A930038C07Rik	372	Grimm	NM_172399
fibronectin type III domain containing 3B	Fndc3b	373	Grimm	
protein phosphatase 1, regulatory (inhibitor) subunit 14B	Ppp1r14b	374	Grimm	NM_008889
myosin Vb	Myo5b	376	Grimm	NM_201600
phosphofructokinase, liver, B-type	Pfkl	377	Grimm	NM_008826
thyroid transcription factor 1	Titf1	378	Grimm	NM_009385
claudin 1	Cldn1	379	Grimm	NM_016674
guanine nucleotide binding protein (G protein), gamma 11	Gng11	380	Grimm	NM_025331
scrapie responsive gene 1	Scrg1	381	Grimm	NM_009136
X-box binding protein 1	Xbp1	382	Grimm	NM_013842
G protein-coupled receptor, family C, group 5, member A	Gprc5a	384	Grimm	NM_181444
phosphoglucomutase 2-like 1	Pgm2l1	387	Grimm	
regulator of G-protein signaling 2	Rgs2	388	Grimm	
cytochrome P450, family 51	Cyp51	2	Greene	NM_020010
ubiquitin carboxy-terminal hydrolase L1	Uchl1	31	Greene	NM_011670
brain derived neurotrophic factor	Bdnf	57	Greene	NM_001048139
pyruvate dehydrogenase E1 alpha 1	Pdha1	119	Greene	NM_008810
phospholipase C, beta 4	PLCB4	232	Greene	NM_013829
lysophospholipase 1	Lypla1	233	Greene	NM_008866
solute carrier family 24 (sodium/potassium/calcium exchanger), membe	Slc24a2	234	Greene	
ATPase, Ca++ transporting, cardiac muscle, slow twitch 2	Atp2a2	235	Greene	NM_009722
calpain, small subunit 1	Capns1	236	Greene	NM_009795
inositol 1,4,5-triphosphate receptor 1	Itpr1	237	Greene	NM_010585
cysteine rich protein 2	Crip2	238	Greene	NM_024223
dynein cytoplasmic 1 intermediate chain 1	Dync1i1	239	Greene	NM_010063
ubiquinol-cytochrome c reductase, Rieske iron-sulfur polypeptide 1	UQCRFS1	240	Greene	NM_025710
ATP synthase, H+ transporting, mitochondrial F1 complex, alpha subunit	Atp5a1	241	Greene	NM_007505
glutamate oxaloacetate transaminase 2, mitochondrial	Got2	242	Greene	NM_010325
pyruvate kinase, muscle	Pkm2	243	Greene	NM_011099
creatine kinase, brain	Ckb	244	Greene	NM_021273
adenylate kinase 1	Ak1	245	Greene	NM_021515
guanine nucleotide binding protein, alpha o	Gnao1	246	Greene	NM_010308
enolase 3, beta muscle	Eno3	247	Greene	NM_007933
dopamine receptor 2	Drd2	248	Greene	NM_010077

calcium channel, voltage-dependent, T type, alpha 1G subunit	Cacna1g	249	Greene	NM_009783
protein kinase C, beta 1	Prkcb1	250	Greene	NM_008855
protein kinase, cAMP dependent regulatory, type II beta	Prkar2b	252	Greene	NM_011158
proprotein convertase subtilisin/kexin type 2	Pcsk2	255	Greene	NM_008792
neuritin 1	Nrn1	256	Greene	NM_153529
monoamine oxidase A	Maoa	257	Greene	NM_173740
GTP cyclohydrolase 1	Gch1	258	Greene	NM_008102
solute carrier family 7 (cationic amino acid transporter, y+ system), mer	Slc7a3	259	Greene	NM_007515
cathepsin D	Ctsd	263	Greene	NM_009983
vesicle-associated membrane protein 2	Vamp2	264	Greene	NM_009497
	Tle1	395	Greene	
	Grb10	396	Greene	
	Tom1l2	397	Greene	
	A230083H22Rik	398	Greene	
calbindin-28K	Calb1	6	Chung	NM_009788
guanosine diphosphate (GDP) dissociation inhibitor 2	Gdi2	18	Chung	NM_008112
longevity assurance homolog 4	Lass4	26	Chung	NM_026058
microtubule-associated protein 1 light chain 3 alpha	Map1lc3a	27	Chung	NM_025735
nuclear receptor interacting protein 3	Nrip3	34	Chung	NM_020610
CD24a antigen	Cd24a	35	Chung	NM_009846
RAB3C, member RAS oncogene family	Rab3c	36	Chung	NM_023852
acyl-CoA synthetase long-chain family member 6	Acsl6	45	Chung	NM_001033597
myosin Va	Myo5a	105	Chung	NM_010864
RAR-related orphan receptor alpha	Rora	134	Chung	
syndecan 2	Sdc2	141	Chung	NM_008304
fibroblast growth factor 1	Fgf1	171	Chung	NM_010197
poliovirus receptor-related 3	Pvrl3	172	Chung	NM_021495
glutamate receptor, ionotropic, NMDA2C (epsilon 3)	Grin2c	173	Chung	NM_010350
zinc finger, DHHC domain containing 2	Zdhhc2	174	Chung	NM_178395
cytochrome P450, family 4, subfamily v, polypeptide 3	Cyp4v3	175	Chung	NM_133969
special AT-rich sequence binding protein 1	Satb1	176	Chung	NM_009122
acetyl-Coenzyme A dehydrogenase, long-chain	Acadl	177	Chung	NM_007381
solute carrier family 25 (mitochondrial carrier, adenine nucleotide transl	Slc25a5	178	Chung	NM_007451
vav 3 oncogene	Vav3	179	Chung	NM_020505
calcium channel, voltage dependent, alpha2/delta subunit 3	Cacna2d3	180	Chung	NM_009785
synuclein, gamma	Sncg	181	Chung	NM_011430

oxysterol binding protein-like 11	Osbpl11	182	Chung	NM_176840
limb expression 1 homolog (chicken)	Lix1	183	Chung	NM_025681
annexin A1	Anxa1	184	Chung	
Ca<2+>dependent activator protein for secretion	Cadps	186	Chung	NM_001042617
RAS-like, family 11, member B	Rasl11b	187	Chung	NM_026878
polymerase (DNA directed), beta	Polb	188	Chung	
cyclin I	Ccni	189	Chung	NM_017367
RNA terminal phosphate cyclase domain 1	Rtcd1	190	Chung	NM_025517
lysosomal-associated protein transmembrane 4B	Laptm4b	192	Chung	NM_033521
cadherin 8	Cdh8	193	Chung	NM_001039154
ganglioside-induced differentiation-associated-protein 1	Gdap1	194	Chung	NM_010267
adenosine kinase	Adk	195	Chung	NM_134079
t-complex-associated-testis-expressed 1-like	Tcte1l	196	Chung	
aspartyl aminopeptidase	Dnpep	197	Chung	NM_016878
regulator of G-protein signaling 8	Rgs8	199	Chung	NM_026380
pre B-cell leukemia transcription factor 3	Pbx3	200	Chung	NM_016768
trophoblast glycoprotein	Tpbg	201	Chung	NM_011627
CDP-diacylglycerol synthase (phosphatidate cytidylyltransferase) 2	Cds2	202	Chung	NM_138651
myocyte enhancer factor 2A	Mef2a	203	Chung	NM_001033713
mitogen activated protein kinase 9	Mapk9	204	Chung	NM_016961
F-box only protein 2	Fbxo2	205	Chung	NM_176848
macrophage migration inhibitory factor	Mif	206	Chung	NM_010798
NADH dehydrogenase (ubiquinone) Fe-S protein 8	Ndufs8	207	Chung	NM_144870
ELOVL family member 6, elongation of long chain fatty acids (yeast)	Elovl6	208	Chung	NM_130450
CD47 antigen (Rh-related antigen, integrin-associated signal transducer)	Cd47	209	Chung	NM_010581
citrate synthase	Cs	210	Chung	NM_026444
phosphatidylinositol transfer protein alpha	Pitpna	211	Chung	NM_008850
homer homolog 2 (Drosophila)	Homer2	212	Chung	NM_011983
RASD family, member 2	Rasd2	213	Chung	XM_204287
thioredoxin domain containing 14	Txndc14	215	Chung	NM_025868
3-oxoacid CoA transferase 1	Oxct1	216	Chung	NM_024188
translocase of outer mitochondrial membrane 20 homolog (yeast)	Tomm20	217	Chung	NM_024214
N-ethylmaleimide sensitive fusion protein attachment protein beta	Napb	218	Chung	NM_019632
amyloid beta (A4) precursor protein-binding, family A, member 2	Apba2	219	Chung	NM_007461
NIMA (never in mitosis gene a)-related expressed kinase 7	Nek7	221	Chung	NM_021605
vacuolar protein sorting 35	Vps35	222	Chung	NM_022997

RAB6, member RAS oncogene family	Rab6	223	Chung	NM_024287
very low density lipoprotein receptor	Vldlr	224	Chung	NM_013703
GTPase activating protein 24	Arhgap24	226	Chung	NM_029270
visinin-like 1	Vsnl1	227	Chung	NM_012038
protein tyrosine phosphatase, non-receptor type 5	Ptpn5	228	Chung	NM_013643
prohibitin 2	Phb2	229	Chung	NM_007531
NADH dehydrogenase (ubiquinone) Fe-S protein 2	Ndufs2	230	Chung	NM_153064
transducin-like enhancer of split 3, homolog of Drosophila E(spl)	Tle3	231	Chung	NM_009389
tachykinin receptor 3	Tacr3	254	Chung	NM_021382
serine protease inhibitor, Kunitz type 2	Spint2	262	Chung	NM_011464
colony stimulating factor 2 receptor, beta 2, low-affinity (granulocyte-ma	Csf2rb2	266	Chung	NM_007781
reticulocalbin 1	Rcn1	267	Chung	NM_009037
procollagen, type XI, alpha 1	Col11a1	268	Chung	
FXYD domain-containing ion transport regulator 6	Fxyd6	269	Chung	NM_022004
calbindin 2	Calb2	270	Chung	NM_007586
MARCKS-like 1	Marcksl1	271	Chung	NM_010807
cholecystokinin	Cck	272	Chung	NM_031161
mesoderm development candiate 2	Mesdc2	273	Chung	NM_023403
RIKEN cDNA 9130213B05 gene	9130213B05Rik	274	Chung	NM_145562
RIKEN cDNA 9130005N14 gene	9130005N14Rik	275	Chung	
huntingtin-associated protein 1	Hap1	276	Chung	NM_010404
SWI/SNF related, matrix associated, actin dependent regulator of chrom	Smarca1	277	Chung	NM_053123
dedicator of cytokinesis 6	Dock6	278	Chung	
CaM kinase-like vesicle-associated	Camkv	279	Chung	NM_145621
isocitrate dehydrogenase 1 (NADP+), soluble	Idh1	280	Chung	NM_010497
nucleobindin 2	Nucb2	281	Chung	NM_016773
ribosomal protein L36a	Rpl36a	282	Chung	NM_019865
lipoprotein lipase	Lpl	339	Chung	NM_008509
adenylate cyclase activating polypeptide 1	Adcyap1	347	Chung	NM_009625
G substrate	Gsbs	385	Chung	NM_011153
cDNA sequence BC005537	BC005537	30	Barrett	NM_024473
coiled-coil domain containing 91	Ccdc91	40	Barrett	NM_025911
cadherin 11	Cdh11	41	Barrett	NM_009866
myeloid/lymphoid or mixed-lineage leukemia (trithorax homolog, Drosop	Mllt11	48	Barrett	NM_019914
acidic ribosomal phosphoprotein P0	Arbp	49	Barrett	NM_007475
chimerin 1	Chn1	62	Barrett	NM_029716

in locus of hypothetical protein D230050A05	D230050A05	68	Barrett	NM_177585
transmembrane protein 30A	Tmem30a	69	Barrett	NM_133718
drebrin 1	Dbn1	70	Barrett	NM_019813
deleted in colorectal carcinoma	dcc	71	Barrett	NM_007831
delta/notch-like EGF-related receptor	Dner	74	Barrett	NM_152915
lute carrier family 10 (sodium/bile acid cotransporter family), member 4	Slc10a4	75	Barrett	NM_173403
eukaryotic translation elongation factor 1 alpha 1	Eef1a1	76	Barrett	NM_010106
fatty acid binding protein 5, epidermal	Fabp5	78	Barrett	NM_010634
coagulation factor II (thrombin) receptor	F2r	79	Barrett	NM_010169
farnesyl diphosphate farnesyl transferase 1	Fdft1	80	Barrett	NM_010191
farnesyl diphosphate synthetase	Fdps	81	Barrett	NM_134469
hepatocyte nuclear factor 3 alpha	foxa1	82	Barrett	NM_008259
four and a half LIM domains 1	fhl1	83	Barrett	NM_001077361
growth associated protein 43	Gap43	84	Barrett	NM_008083
heat shock factor binding protein 1	Hsbp1	87	Barrett	NM_024219
iduronate 2-sulfatase	Ids	88	Barrett	NM_001038990
limbic system-associated membrane protein	Lsamp	94	Barrett	NM_175548
itogen activated protein kinase 1	Mapk11	96	Barrett	NM_011161
microtubule-associated protein tau	Mapt	97	Barrett	NM_001038609
matrin 3	Matr3	99	Barrett	NM_010771
mortality factor 4 like 2	Morf4l2	101	Barrett	NM_019768
ybe mortality factor 4 like 1	Morf4l1	102	Barrett	NM_001039147
NADH dehydrogenase (ubiquinone) 1 alpha subcomplex 10	Ndufa10	108	Barrett	NM_024197
n-myc downstream regulated 3	Ndrg3	109	Barrett	NM_013865
neural precursor cell expressed, developmentally down-regulted gene 4	Nedd4	110	Barrett	NM_010890
nicolin 1	Nicn1	111	Barrett	NM_025449
nuclear receptor subfamily 4, group A, member 2	Nr4a2	112	Barrett	NM_013613
TrkB, neurotrophic tyrosine kinase, receptor, type 2	Ntrk2	113	Barrett	NM_001025074
neurexin III	Nrxn3	115	Barrett	NM_172544
progesterone receptor membrane component 1	Pgrmc1	120	Barrett	NM_016783
phosphoglycerate kinase 1	Pgk1	121	Barrett	NM_008828
PQ loop repeat containing 1	Pqlc1	124	Barrett	NM_025861
protein phosphatase 3, regulatory subunit B, alpha isoform (calcineurin I	Ppp3r1	125	Barrett	NM_024459
RNA binding motif protein 4	Rbm4	130	Barrett	NM_009032
RNA binding motif protein 18	Rbm18	131	Barrett	NM_026434
ring finger protein 7	Rnf7	133	Barrett	NM_011279

ataxin 10	Atxn10	138	Barrett	NM_016843
secretogranin II	Scg2	139	Barrett	NM_009129
succinate dehydrogenase complex, subunit B, iron sulfur (Ip)	Sdhb	143	Barrett	NM_023374
single-stranded DNA binding protein 2	Ssbp2	153	Barrett	NM_024186
translocase of inner mitochondrial membrane 10 homolog (yeast)	Timm10	159	Barrett	NM_013899
serine incorporator 1	Serinc1	160	Barrett	NM_019760
ubiquitin-conjugating enzyme E2B, RAD6 homology (S. cerevisiae)	Ube2b	165	Barrett	NM_009458
valosin containing protein	Vcp	167	Barrett	NM_009503
tyrosine 3-monooxygenase/tryptophan 5-monooxygenase activation pro	Ywhae	168	Barrett	NM_009536
tyrosine 3-monooxygenase/tryptophan 5-monooxygenase activation pro	Ywhab	169	Barrett	NM_018753
SH3-binding domain glutamic acid-rich protein like	Sh3bgrl	220	Barrett	NM_019989
chaperonin subunit 5 (epsilon)	Cct5	367	Barrett	NM_007637
	5730410E15Rik	391	Barrett	
ADP-ribosylation factor-like 5A	Arl5a	4		
asparagine-linked glycosylation 2 homolog	Alg2	5		NM_019998
calcium channel, voltage-dependent, beta 2 subunit	Cacnb2	7		NM_023116
carbonic anhydrase 4	Car4	8		NM_007607
dickkopf homolog 3	Dkk3	10		NM_015814
dystrophia myotonica-containing WD repeat motif	Dmwd	11		NM_010058
Eph receptor A6	Epha6	12		NM_007938
expressed in non-metastatic cells 1, protein	Nme1	13		NM_008704
glutamate receptor, ionotropic, kainate 3	Grik3	14		XM_131647
glutamate receptor, ionotropic, kainate 3	Grik3	15		XM_131647
heme oxygenase (decycling) 2	Hmox2	17		NM_010443
histone 3, H2a	Hist3h2a	19		NM_178218
HIV-1 tat interactive protein 2, homolog (human	Htatip2	20		NM_016865
mannoside acetylglucosaminyltransferase 5, isoenzyme B	Mgat5b	21		NM_172948
RIKEN cRIKEN cDNA 9630033F20 gene DNA 9630033F20 gene	9630033F20Rik	25		
ribosomal protein L11	Rpl11	28		NM_025919
huntingtin interacting protein 2	Hip2	29		NM_016786
zinc finger, MYND domain containing 11	Zmynd11	32		NM_144516
potassium inwardly-rectifying channel, subfamily J, member 6	Kcnj6	33		NM_001025584
4-aminobutyrate aminotransferase	Abat	43		
solute carrier family 6 (neurotransmitter transporter, dopamine), membe	Slc6a3 (or DAT)	148		NM_010020
	Timm8a	162		
SH3-binding kinase 1	Sbk1	185		NM_145587

myelocytomatosis oncogene	Myc	285		NM_010849
nerve growth factor receptor (TNFR superfamily, member 16)	Ngfr	286		NM_033217
nucleoporin 93	Nup93	287		NM_172410
protocadherin alpha 10	Pcdha10	288		NM_009961
protein phosphatase 2, regulatory subunit B (B56), delta isoform	Ppp2r5d	289		NM_009358
receptor accessory protein 1	Reep1	290		NM_178608
ribophorin I	Rpn1	291		NM_133933
septin 5	Sept5	293		NM_213614
solute carrier family 35, member C2	Slc35c2	294		NM_144893
SET domain containing (lysine methyltransferase) 7	Setd7	295		NM_080793
SNF related kinase	Snrk	296		NM_133741
single stranded DNA binding protein 4	Ssbp4	297		NM_133772
suppressor of Ty 5 homolog (S. cerevisiae)	Supt5h	298		NM_013676
serine/threonine kinase 32C	Stk32c	299		NM_021302
testis enhanced gene transcript	Tegt	301		NM_026669
transmembrane protein 50B	Tmem50b	302		NM_030018
tousled-like kinase 1	Tlk1	303		NM_172664
transformation related protein 53 binding protein 1	Trp53bp1	304		NM_013735
tetraspanin 6	Tspan6	305		
