# Supplementary material for: Linkage of cDNA expression profiles of mesencephalic dopaminergic neurons to a genome-wide in situ hybridization database
Source: Mol Neurodegener. 2009 Jan 29;4:6. doi: 10.1186/1750-1326-4-6 (PMC2637272; doi:10.1186/1750-1326-4-6)
Supplement: Additional file 2 — Number of genes, identified by six screens, aimed at determining the expression profile of mesDA neurons. Total number of genes, as well as the number of transcripts, expressed in SNpc or VTA, detectable by ISH, within the ABA database is shown. Efficiency of each screen in identifying transcripts, which show above-background expression within either or both of the two regions, according to ABA is between 24–38%, while 56% of the genes from combination of all screens show expression. [file 1750-1326-4-6-S2.pdf]

	TOTAL	SN/VT	% of total	Barrett	Chung	Greene	Grimm	Steward	Thuret
Barrett	199	57	29%	**57**	3	4	3	11	1
Chung	383	95	25%		**95**	12	15	4	4
Greene	137	51	37%			**51**	10	3	0
Grimm	412	100	24%				**100**	3	2
Steward	273	76	28%					**76**	2
Thuret	119	29	24%						**29**
All Screen	**606**	**341**	**56%**						
