# Supplementary material for: Linkage of cDNA expression profiles of mesencephalic dopaminergic neurons to a genome-wide in situ hybridization database
Source: Mol Neurodegener. 2009 Jan 29;4:6. doi: 10.1186/1750-1326-4-6 (PMC2637272; doi:10.1186/1750-1326-4-6)
Supplement: Additional file 3 — Compilation of gene symbols, identified by Neuroblast function of ABA. The template genes Foxa2, En1, Th, Vmat2 and Dat, used for neurblast, are expressed and have defined functions in neurotransmitter phenotype or transcriptional regulation of survival and maintenance of dopaminergic neurons. 250 genes were listed as having similar expression patterns to each template. Repetitions are eliminated from the final list. [file 1750-1326-4-6-S3.pdf]

No.	Gene Symbol	101	Cacng5	202	Dpysl5	303	Gyg	404	Mast4	505	Ppap2b	606	Slc6a9
1	Abat	102	Cacng7	203	Drd2	304	Hap1	405	mCG140667	506	Ppfibp1	607	Slc8a3
2	Abhd3	103	Cacybp	204	Dscr1l1	305	Hars2	406	Mdfi	507	Pqlc1	608	Slc9a9
3	Ablim3	104	Cadps2	205	Dusp10	306	Hcn2	407	Megf10	508	Prdx1	609	Slco3a1
4	Acat2	105	Cage1	206	Dusp5	307	Hcrtr1	408	Megf11	509	Prkcd	610	Sncg
5	Ache	106	Calb2	207	Ece2	308	Hdc	409	Meis1	510	Prkg1	611	Socs6
6	Acsbg1	107	Calcb	208	Ecel1	309	Hes3	410	Mesdc2	511	Prmt2	612	Sod1
7	Acsl3	108	Calcr	209	Edc3	310	Hip1	411	Mfap5	512	Prokr2	613	Sp8
8	Actr10	109	Calml3	210	Edg1	311	Hmgcs1	412	Mlstd1	513	Prrg2	614	Sparc
9	Acyp2	110	Capn2	211	Ednrb	312	Hod	413	Mm.271301	514	Psmd5	615	Spata13
10	Adamts19	111	Car10	212	Eif5a2	313	Homer2	414	Mrg1	515	Psme1	616	Spp1
11	Adamts2	112	Car2	213	Elavl2	314	Hrasls3	415	Mrg2	516	Psme2	617	Sptlc2
12	Adamts4	113	Car4	214	Elovl5	315	Hspa4l	416	Mrpl39	517	Pstpip1	618	Sqle
13	Adarb1	114	Car7	215	Elovl6	316	Icmt	417	Msi2	518	Ptgds	619	Srgap1
14	Adarb2	115	Cart	216	En2	317	Idh1	418	Msto1	519	Ptpn2	620	Srprb
15	Adcy4	116	Casq2	217	Endod1	318	Igfbp5	419	Mtap4	520	Ptpn4	621	Ssbp2
16	Adcy8	117	Casr	218	Entpd3	319	Igsf3	420	Mtrr	521	Ptprm	622	St8sia1
17	Adcyap1	118	Cast	219	Eomes	320	Il13ra1	421	Mtss1	522	Ptpro	623	Stac
18	Adcyap1r1	119	Ccdc91	220	Ephb1	321	Il16	422	Mum1l1	523	Ptprr	624	Stat5b
19	Adipor1	120	Cd81	221	Epn3	322	Impact	423	Myt1	524	Ptprz1	625	Steap2
20	AF529169	121	Cda	222	Erbb4	323	Impg2	424	Nab1	525	Ptrf	626	Stk17b
21	Agc1	122	Cdc42ep1	223	Ero1lb	324	Inhba	425	Ndn	526	Pvalb	627	Stmn3
22	Agt	123	Cdgap	224	Esrrg	325	Inpp4b	426	Ndrg2	527	Pvr	628	Stx3
23	Agtr1b	124	Cdh15	225	Etv1	326	Inpp5a	427	Ndufb8	528	Pygb	629	Sv2c
24	Agxt2l1	125	Cdh4	226	Evi5	327	Insig1	428	Ndufs1	529	Qdpr	630	Syne2
25	AI427515	126	Cdh8	227	Exph5	328	Iqsec3	429	Nefh	530	Rab37	631	Syt10
26	AI450948	127	Cdkn1a	228	F2r	329	Irs4	430	Nell1	531	Rab3c	632	Syt6
27	AI836003	128	Cdkn2b	229	Fabp5	330	Isoc1	431	Neurl	532	Raf1	633	Syt9
28	Akap12	129	Cdr2	230	Fabp7	331	Itga3	432	Neurod1	533	Rasa2	634	Tacr3
29	Akap13	130	Cerk	231	Fbn2	332	Itgb1	433	Nfil3	534	Rasa4	635	Tal1
30	Akap2	131	Chd7	232	Fbxo9	333	Itgb1bp1	434	Nfyc	535	Rassf8	636	Tbl3
31	Akr1b3	132	Chml	233	Fdft1	334	Itih3	435	Ngb	536	Raver2	637	TC1410973
32	Aldh1a1	133	Chn2	234	Fdps	335	Jam2	436	Nhlh2	537	Rax	638	TC1517215
33	Aldh1l1	134	Chrm2	235	Fgd3	336	Jup	437	Nhlrc1	538	Rbed1	639	TC1563370
34	Aldh5a1	135	Chrm5	236	Fgf13	337	Kbtbd3	438	Nifun	539	Rbpms2	640	TC1563688
35	Aldoc	136	Chrna3	237	Flt3l	338	Kcnb2	439	Nmb	540	Rcn2	641	TC1568600
36	Alg2	137	Chrna4	238	Fmo1	339	Kcnc1	440	Nola3	541	Reln	642	Tcf2
37	Alkbh6	138	Chrna5	239	Fn1	340	Kcnc2	441	Nova1	542	Resp18	643	Tex261
38	Alox8	139	Chrna6	240	Fndc5	341	Kcnd2	442	Npffr2	543	Rfk	644	Tgfb1
39	Amigo2	140	Chrnb3	241	Fos	342	Kcnd3	443	Npr1	544	Rfx4	645	Th
40	Amot	141	Chst8	242	Fryl	343	Kcng4	444	Nrg1	545	Rgs3	646	Thra
41	Amotl1	142	Cib2	243	Fst	344	Kcnip1	445	Nrip3	546	Rgs8	647	Tiam1
42	Ank1	143	Clcn5	244	Fstl5	345	Kcnip4	446	Nrsn2	547	Rhobtb3	648	Tm2d3
43	Ankrd24	144	Cldn11	245	Fts	346	Kcnk1	447	Nt5m	548	Rnd2	649	Tm2d3

44	Ankrd38	145	Clgn	246	Fut8	347	Kcnk13	448	Ntn1	549	Rnf13	650	Tm6sf1
45	Anln	146	Clic4	247	Fzd8	348	Kcns3	449	Ntsr1	550	Rnh1	651	Tm6sf1
46	Anxa2	147	Clmn	248	Gaa	349	Kctd9	450	Nxn	551	Rorc	652	Tmed3
47	Apba1	148	Clptm1l	249	Gabra6	350	Kifc3	451	Nxph4	552	Rpl11	653	Tmem130
48	Apbb2	149	Cmbl	250	Gabre	351	Kit	452	Og9x	553	Rps12	654	Tmem163
49	Apc	150	Cnksr3	251	Gabrq	352	Kitl	453	Opn3	554	Rps5	655	Tmem16f
50	Aqp2	151	Cnp1	252	Gad1	353	Klhl1	454	Optn	555	Rragd	656	Tmem22
51	Aqp4	152	Cntn6	253	Gad2	354	Klhl4	455	Osbpl9	556	Rwdd2	657	Tmem29
52	Arhgap12	153	Cntnap3	254	Gal	355	Klk6	456	Ostf1	557	S100a10	658	Tmem33
53	Arhgap5	154	Cntnap4	255	Galnt10	356	L1cam	457	P2ry6	558	S100a16	659	Tmem41a
54	Arhgdig	155	Col11a1	256	Galnt13	357	Lamb1-1	458	P4ha2	559	S100b	660	Tmem47
55	Arl10	156	Col18a1	257	Galnt6	358	Lancl3	459	Pacs2	560	Sacm1l	661	Tmem64
56	Arl2	157	Col24a1	258	Gata3	359	Lcat	460	Pacsin2	561	Samd14	662	Tmie
57	Arl5a	158	Col27a1	259	Gatm	360	Lef1	461	Pappa2	562	Sap30l	663	Tnc
58	Armc2	159	Col5a3	260	Gbe1	361	Lgi3	462	Paqr8	563	Sash1	664	Tnfaip8
59	Arntl	160	Col9a1	261	Gcg	362	Lgr5	463	Park2	564	Sc4mol	665	Tnrc6a
60	Arx	161	Cops4	262	Gch1	363	Lhfp	464	Pax6	565	Sc5d	666	Tns1
61	Asb4	162	Coro2b	263	Gchfr	364	Lhx1	465	Pax7	566	Scarb2	667	Tob2
62	Ascl1	163	Cox6c	264	Gdf11	365	Lhx5	466	Pbx3	567	Scd2	668	Tpd52l1
63	Astn2	164	Cpne2	265	Gdpd2	366	Limk1	467	Pcbp3	568	Scd3	669	Tph2
64	Asxl3	165	Creg1	266	Gfra1	367	Lip1	468	Pcdh18	569	Scg2	670	Trh
65	Atbf1	166	Crhr1	267	Ghsr	368	Lix1	469	Pcdh21	570	Scube2	671	Trim36
66	Atf3	167	Crhr2	268	Gja1	369	Lmx1b	470	Pcdh9	571	Sdc2	672	Tshz1
67	Atic	168	Csdc2	269	Gja7	370	Lnx2	471	Pcp2	572	Sema3c	673	Tspan11
68	Atp10a	169	Csk	270	Gja9	371	LOC244958	472	Pcsk1n	573	Sema3d	674	Tspan12
69	Atp11c	170	Cspg2	271	Gldc	372	LOC270764	473	Pcsk6	574	Sema3f	675	Tspan15
70	Atp8a2	171	Ctbp2	272	Glra1	373	LOC381076	474	Pdcl	575	Sema4d	676	Tspan2
71	AU040377	172	Cthrc1	273	Glra3	374	LOC432637	475	Pde4d	576	Sema4g	677	Tspan6
72	AW049765	173	Ctnnbip1	274	Glul	375	LOC433088	476	Pde9a	577	Sema6a	678	Ttbk2
73	B3gnt5	174	Ctsl	275	Gm1335	376	LOC433093	477	Pdxk	578	Sema6c	679	Tubb4
74	B4galt1	175	Cygb	276	Gm69	377	LOC433228	478	Pdzd8	579	Sema6d	680	Tulp4
75	B4galt5	176	Cyp27a1	277	Gng11	378	LOC433254	479	Peg10	580	Serpinb1c	681	Txnrd3
76	Bace2	177	Cyp51	278	Gng13	379	LOC433258	480	Phactr2	581	Serpine2	682	Ube1l2
77	Baiap3	178	D0H4S114	279	Gng2	380	LOC433485	481	Phactr4	582	Sez6l2	683	Ublcp1
78	BC004044	179	D11Ertd333e	280	Gng4	381	LOC545810	482	Phf6	583	Sgpp2	684	Ugt8a
79	BC025816	180	D8Ertd82e	281	Gpc3	382	Loh11cr2a	483	Pias4	584	Sh3bgrl2	685	Unc5b
80	BC029169	181	Dad1	282	Gpc6	383	Loxl1	484	Pib5pa	585	Sh3d19	686	Unc5c
81	BC030477	182	Dbh	283	Gpr165	384	Lpin1	485	Pkia	586	Shb	687	Utp14b
82	BC031353	183	Dbi	284	Gpr171	385	Lrig1	486	Pknox1	587	Sla	688	Vangl1
83	BC051227	184	Ddc	285	Gpr37l1	386	Lrp8	487	Plcb4	588	Slc10a4	689	Vat1
84	BC054438	185	Ddc8	286	Gpr56	387	Lrrc1	488	Plcd1	589	Slc12a8	690	Veph1
85	BC057022	186	Ddt	287	Gpr98	388	Lrrc3b	489	Plcl1	590	Slc17a6	691	Vim
86	Bcat1	187	Decr1	288	Gprc5b	389	Lrrc55	490	Plekha2	591	Slc17a8	692	Vipr2
87	Bckdhb	188	Depdc6	289	Gpx3	390	Lrsam1	491	Plekha7	592	Slc18a2	693	Vps39

88	Bmp5	189	Dexi	290	Grb10	391	Ltbp3	492	Plekhg1	593	Slc18a3	694	Wnt10a
89	Brunol6	190	Dhcr24	291	Grid2	392	Lxn	493	Plekhh2	594	Slc20a2	695	Wnt10b
90	Btbd11	191	Dip3b	292	Grid2ip	393	Lypla1	494	Plp1	595	Slc22a3	696	Wnt3
91	Btbd3	192	Dlat	293	Grin2c	394	Lzp-s	495	Pltp	596	Slc32a1	697	Wrb
92	Btg1	193	Dlk1	294	Grinl1a	395	Mad1l1	496	Plxdc1	597	Slc34a1	698	Zcchc12
93	Btg2	194	Dlx1	295	Grm1	396	Maged2	497	Plxdc2	598	Slc38a1	699	Zfhx2as
94	C1ql2	195	Dlx6os1	296	Grm4	397	Magel2	498	Plxnb2	599	Slc38a3	700	Zfhx4
95	C85492	196	Dnahc11	297	Grp	398	Manba	499	Pmfbp1	600	Slc41a3	701	Zfp521
96	C85492	197	Dnajc12	298	Gsn	399	Maoa	500	Pnpo	601	Slc5a7	702	Zic1
97	Cables2	198	Dnm3	299	Gstm4	400	Maob	501	Podxl2	602	Slc6a11	703	Zim1
98	Cacna2d2	199	Doc2g	300	Gucy2c	401	Map2k5	502	Pogk	603	Slc6a2		
99	Cacnb2	200	Dock6	301	Gucy2f	402	Map2k6	503	Pogz	604	Slc6a3		
100	Cacng4	201	Dpysl3	302	Guk1	403	Masp1	504	Pon3	605	Slc6a4		
