# Supplementary material for: Linkage of cDNA expression profiles of mesencephalic dopaminergic neurons to a genome-wide in situ hybridization database
Source: Mol Neurodegener. 2009 Jan 29;4:6. doi: 10.1186/1750-1326-4-6 (PMC2637272; doi:10.1186/1750-1326-4-6)
Supplement: Additional file 4 — Comparison of the list of genes from Neuroblast and genome-wide screens. The comparison of the two lists from additional files 1 &2 show that a fraction of the genes from the six screens, considered by this study can be acquired, using the Neuroblast function. [file 1750-1326-4-6-S4.pdf]

No.	Symbol						
1	Abat	18	Chrna4	36	Homer2	54	Rcn2
1	Abat	19	Chrnb3	37	Idh1	55	Rgs8
2	Acsl3	20	Col11a1	38	Impact	56	Rpl11
3	Adcyap1	21	Ddc	39	Kcnd3	57	Sacm1l
4	Akr1b3	22	Dock6	40	Lix1	58	Scg2
5	Aldh1a1	23	Drd2	41	Lrrc3b	59	Sdc2
6	Alg2	24	Elovl6	42	Lypla1	60	Sema6d
7	Amotl1	25	Erbb4	43	Maoa	61	Slc10a4
8	Arl5a	26	Fabp5	44	Mesdc2	62	Slc18a2
9	Atbf1	27	Fdft1	45	Mtap4	63	Slc6a3
10	Cacnb2	28	Fdps	46	Nrip3	64	Sncg
11	Cadps2	29	Gbe1	47	Pbx3	65	Ssbp2
12	Calb2	30	Gfra1	48	Pkia	66	Tacr3
13	Calm3	31	Gng11	49	PLCB4	67	Th
14	Car4	32	Gpx3	50	Plekha7	68	Tmie
15	Casr	33	Grb10	51	Pqlc1	69	Tshz1
16	Ccdc91	34	Grin2c	52	Prmt2	70	Tspan6
17	Cdh8	35	Hap1	53	Rab3c		
